# Cytogenetic and molecular detection of a rare unbalanced Y;3 translocation in an infertile male: A case report

**DOI:** 10.1097/MD.0000000000020863

**Published:** 2020-06-26

**Authors:** Shu Deng, Hongguo Zhang, Xiangyin Liu, Fagui Yue, Yuting Jiang, Shibo Li, Ruizhi Liu, Qi Xi

**Affiliations:** a Center for Reproductive Medicine, Center for Prenatal Diagnosis, First Hospital; b Jilin Engineering Research Center for Reproductive Medicine and Genetics, Jilin University, Changchun, China; c Department of Pediatrics, University of Oklahoma Health Sciences Center, Oklahoma City, USA.

**Keywords:** karyotype-phenotype correlation, severe oligozoospermia, unbalanced translocation, Y-autosome

## Abstract

**Introduction:**

The infertile male individuals carrying the Y-autosome translocations are seldom reported in clinic. Herein, we described a severe oligozoospermic male with rare unbalanced Y;3 translocation transmitted through 3 generations.

**Patient concerns:**

A 33-year-old Chinese male was referred for infertility consultation in our center after 10 years’ primary infertility. He was diagnosed as severe oligozoospermia according to the semen analysis.

**Diagnosis:**

G-banding analysis initially described the karyotype as 46, XY, add (3) (p26) for the patient, and his wife's karyotype was 46, XX. The chromosomal microarray analysis identified 3.81Mb and 0.29Mb duplications in Yq11.223q11.23 and Yq12, separately. No deletions were detected in azoospermia factors (AZF)a, AZFb and AZFc. Fluorescence in situ hybridization analysis further confirmed the existence of sex-determining region Y gene and verified that Yq12 was translocated to the terminal short arm of chromosome 3(3p26).

**Interventions:**

The couple chose intracytoplasmic sperm injection to get their offspring. The wife underwent amniocentesis for cytogenetic analysis but suffered termination of pregnancy due to premature rupture of membranes.

**Outcomes:**

The karyotype of the patient was finally described as 46, X, der(3)t(Y;3)(q11.22;p26). His father and the aborted fetus showed the same karyotypes as the patient.

**Conclusion:**

Our study not only enriched the karyotype-phenotype correlation of Y-autosome translocation, but also strengthened the critical roles of molecular genetic techniques in identifying the chromosomal breakpoints and regions involved.

## 1 Introduction

Y-autosome translocations are rare chromosomal abnormalities which are associated with male infertility.^[1–3]^ The incidence of Y-autosome translocations in general population was about 1/2000.^[4]^ Till now, more than 100 cases have been described.^[5]^ In most cases with balanced Y-autosome translocations, the distal heterochromatic part of the Y chromosome was translocated to the short arm of an acrocentric chromosome.^[6]^ The common translocation form happened between the Yq heterochromatin and 15p, which usually would not affect the fertility in the carriers.^[7]^ However, rare translocations of the euchromatic part of the Y chromosome were frequently associated with azoospermia.^[8]^ The karyotype-phenotype correlation remains unclear for it depends on not only the breakpoints of chromosome Y but also the involved autosomal regions.^[9]^

Herein, we described a severe oligozoospermic male presenting paternally inherited der(3) resulting from the unbalanced translocation between Yq11 and 3p26.

## 2 Case report

A 33-year-old Chinese male was referred for infertility consultation in our center after ten years’ primary infertility. His height was 182 cm and weight was 77 kg. The development/growth of penis was normal. And the left and right testicular volume is about 12 mL separately. Moreover, no other physical abnormalities were observed. A series of routine examinations were conducted. Semen analysis and levels of sex hormones were listed in Table 1. The male was finally diagnosed as severe oligozoospermia according to the semen routine examination.^[10]^ Our study protocol was approved by the Ethics Committee of the First Hospital of Jilin University (No.2016–430), and the informed written consents were obtained from the patient and his family members for publication of this case report and accompanying images.

**Table 1:**
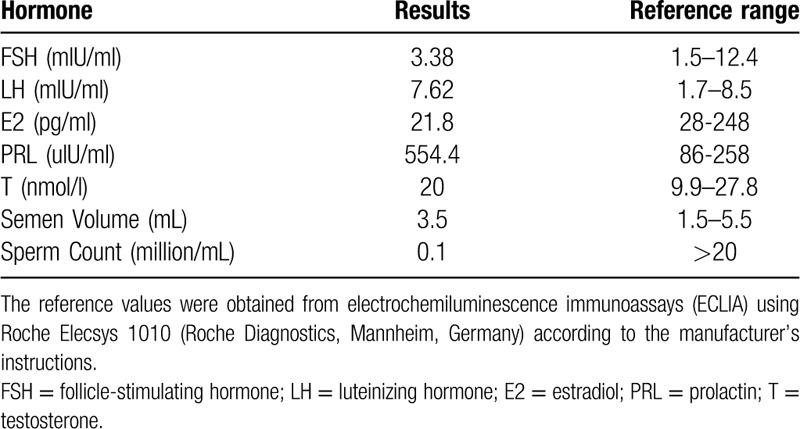
Semen analysis and levels of sex hormones.

## 3 Material and methods

### 3.1 Karyotype analysis

Chromosomal karyotypic analysis were performed on cultured peripheral blood cells and aminotic fluid cells according to the standard cytogenetic protocol. Twenty metaphases were analyzed for the patient and his family members. We described their karyotypes according to the International System for Human Cytogenetic Nomenclature 2016 nomenclature.^[11]^

### 3.2 Chromosomal microarray analysis(CMA)

The DNA was extracted from 5 mL peripheral blood cells and 10 mL of uncultured amino fluid cells using QIAamp DNA Mini kit (Qiagen, Hilden, Germany). The SNP array analysis was carried out using Human CytoSNP-12 BeadChip (Illumina, San Diego, CA). The collected image data were analyzed according to Illumina's Genome Studio software and the results were analyzed through the Database of Chromosomal Imbalance and Phenotype in Humans using Ensemble Resources (DECIPHER), database of genomic variants, Online Mendelian Inheritance in Man, National Center for Biotechnology Information, and so on.^[12]^

### 3.3 Azoospermia factors (AZF) microdeletion analysis

Microdeletions in AZF region were detected using polymerase chain reaction (PCR) technique. Specific sequence-tagged sites(STS) were mapped in the AZF region, including SY84 and SY86 for AZFa, SY27, SY134 and SY143 for AZFb, SY152,SY157, SY254 and SY255 for AZFc.^[13]^

### 3.4 Fluorescence in situ hybridization (FISH) analysis

FISH analysis specifific for chromosome Y was performed on metaphase slides for the patient according to the manufacturer's standard protocol (CytocellTechnologies, Cambridge). The detecting probes are as follows: red labeled sex-determining region Y (SRY) probe presenting 2 non-overlapping probes, green labeled probe for DYZ1.

## 4 Results

**Figure 1:**
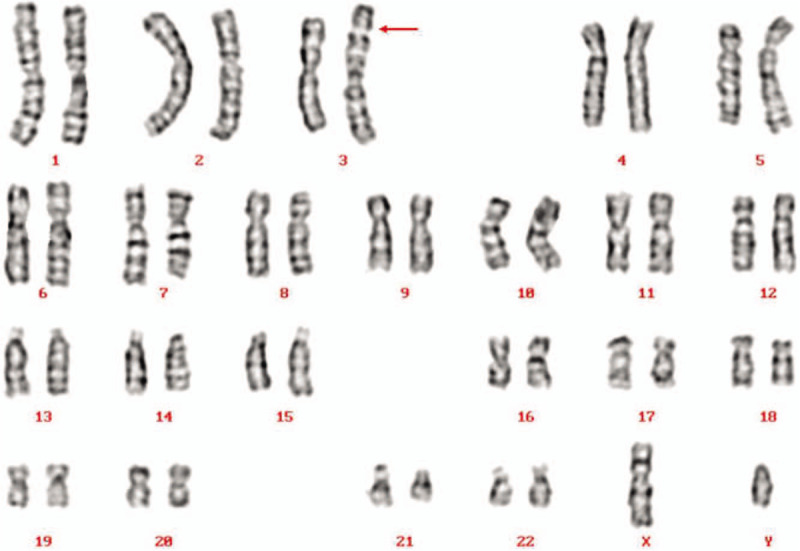
Karyotype of the patient identified by GTG banding technique. Arrow indicated the der(3).

**Figure 2:**
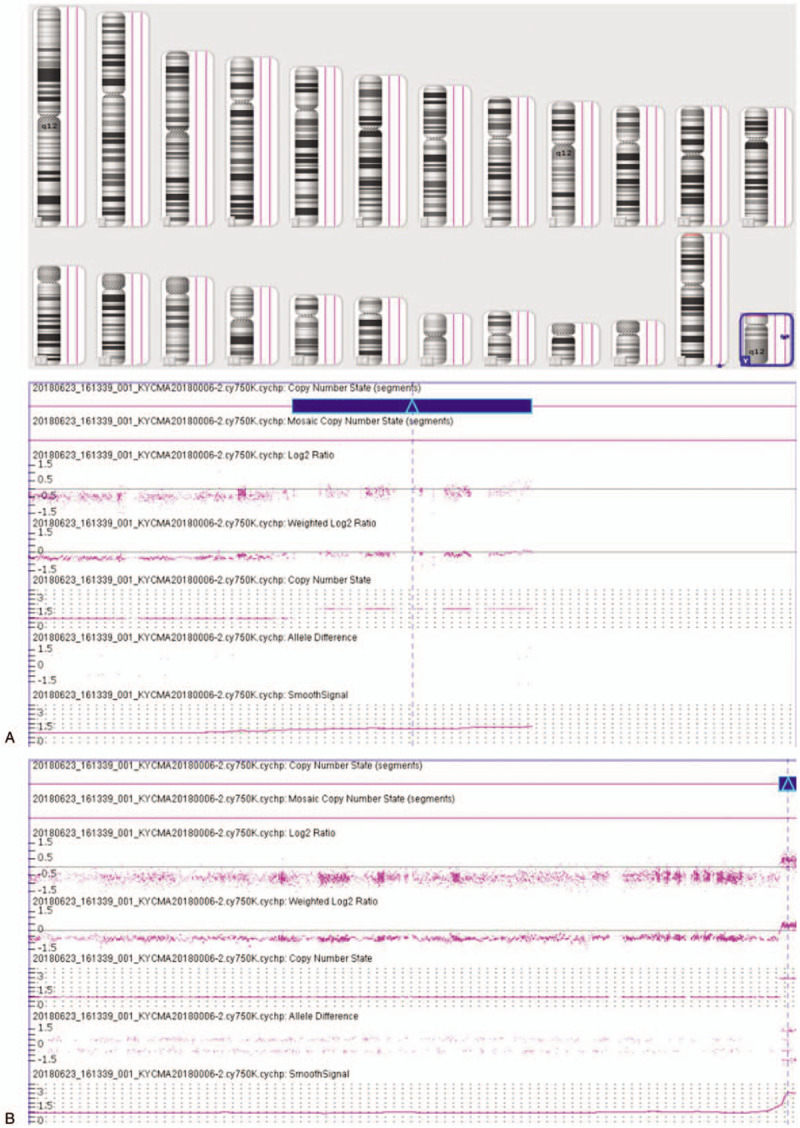
CMA array on peripheral blood depicted Yq11.223q11.23 duplication (A) and Yq12 duplication (B). CMA = chromosomal microarray analysis.

**Figure 3:**
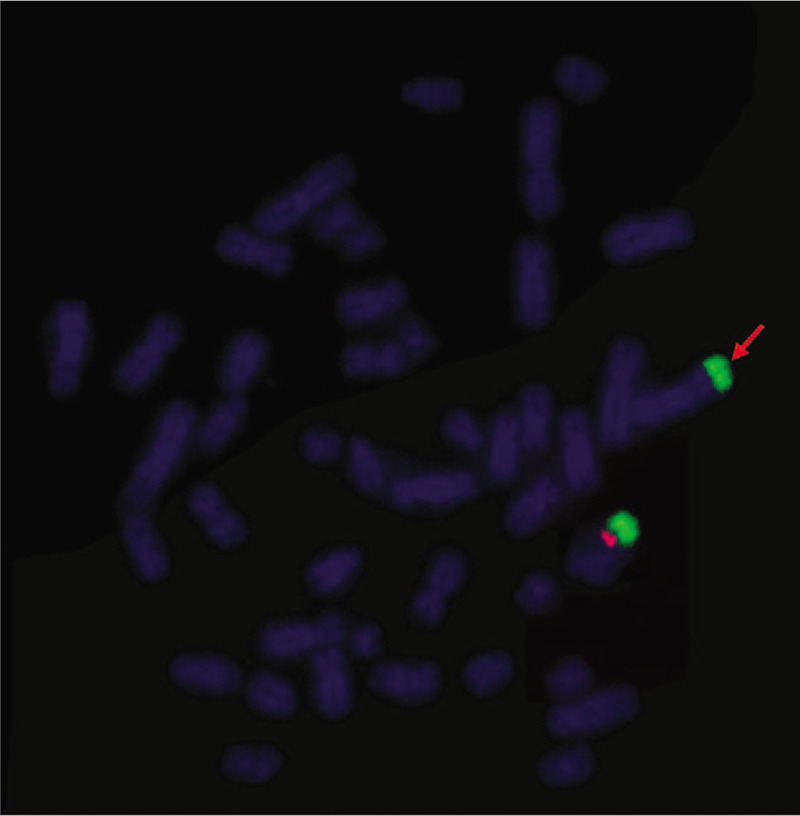
Metaphase-FISH results of SRY probe and Yq12: SRY signal (red) and Yq12 (green). Arrow indicated gains of Yq12. FISH = fluorescence in situ hybridization, SRY = sex-determining region.

G-banding analysis initially described the karyotype as 46, XY,add(3)(p26) for the patient (Fig. 1) and his wife’ karyotype was 46,XX. Then CMA was applied to characterize the add (3) for detail and the results were as follows: arr[hg19] Yq11.223q11.23(24,987,791-28,799, 654)x2; arr[hg19] Yq12(59,046,967-59,336,104)x3 (Fig. 2), which illustrated that there existed 3.81Mb and 0.29Mb duplications in Yq11.223q11.23 and Yq12, separately. Subsequently, FISH using SRY probe and Yq12 was applied for further verification. The FISH results inferred that there were 1 SRY signal and 1 Yq12 signal in the normal chromosome Y, with another Yq12 signal attached to the terminal of chromosome 3 (Fig. 3). To further confirm whether the add (3) was inherited, we recalled the patient's parents back for chromosomal karyotypic analysis. The results showed that the patient got the der (3) from his father, which could be described as unbalanced Y;3 translocation: Yq11.223q12 was translocated to the terminal short arm of chromosome 3 (3p26). Based upon the analysis above, the karyotype of the patient was finally defined as 46, XY, der(3)t(Y;3)(q11.22;p26). According to genetic counseling, the couple chose intracytoplasmic sperm injection to get their offspring. Then the wife underwent amniocentesis for cytogenetic analysis at 17 weeks of gestation, and the fetus was found to get the same der (3) from the patient. But unfortunately the pregnant women suffered spontaneous abortion due to premature rupture of membranes at 23 weeks.

## 5 Discussion

We described a severe oligozoospermic male with an unbalanced paternally inherited t(Y;3) translocation through 3 generations. This Y;3 translocation was initially delineated as a 46, XY,add(3)(p26) through conventional karyotype. Afterwards the CMA and FISH further characterize the add(3) as follows: Yq11.223q12 was translocated to the terminal of chromosome 3p26. To our best knowledge, this unbalanced Y;3 translocation was not reported before.

Compared with translocations between autosomes, sex-autosome translocations shows much stronger effects on fertility than autosome-autosome translocations.^[8]^ The translocations involving sex chromosomes and autosomes are usually associated with azoospermia and divided into 3 categories: Y-autosome, X-autosome and X–Y translocation.^[14]^ Among the 3 groups, Y-autosome translocations are involved in normal and abnormal spermatogenesis.^[3]^ For males with unbalanced Y-autosome translocations, they often present a wide range of clinical phenotypes, such as mental retardation and genital anomalies.^[8,15]^

The reports on Y-autosome translocations involving chromosome 3 were limited. Gonzales et al^[16]^ reported an azoospermic male with balanced reciprocal translocation t(Y;3)(q11.2q12) with normal phenotypes. And they reckoned that the integrity of Y chromosome was probably necessary for normal meiotic process and more important than dosage effect due to complete or partial disomy of Y chromosome. In addition, the CMA results showed a 3.81Mb duplication of q11.223q11.23, overlapping with partial the AZF regions, which was located in Yq11 euchromatin. As is known, microdeletion or complete loss of AZF regions can lead to male infertility.^[17–19]^ Since the patient in our study presented a normal and intact chromosome Y without deletion of AZF loci, which might indicate that the 2 interpretations above were not suitable to explain his impaired spermatogenesis. The breakpoint of the Y chromosome was in Yq12 in fertile males while the breakpoint was assumed to be in distal Yq11 in sterile males,^[8]^ which catered for the presentations in our patient. When the azoospermia factor (AZF) regions are affected by chromosomal translocation, it would cause azoo- or severe oligozoospermia to a great extent.^[6]^ The pseudoautosomal region (PAR) of Y chromosome is essential for the correct pairing of the sex chromosomes. PAR1 is located on the telomere of Xp/Yp and PAR2 is located on the telomere of Xq/Yq.^[20]^ Based upon the literature on Y-autosome translocations, we speculated that the extra duplication involving PAR regions might delay or disturb the the initiation of X and Y pairing, leading to the arrest of meiosis stage I, which would result in azoospermia or severe oligoasthenospermia.^[21]^ In addition, extra copies of AZF region might also be a contributing factor for spermatogenic failure.^[22]^

For the cases with Y-autosome translocations, the combined application of chromosomal karyotyping and FISH is the main approach in detecting this chromosomal anomaly. Besides, the utilization of CMA also plays a critical role in detecting the chromosomal microscopic imbalance, which would offer more detailed elaborations in delineating the possible reasons for infertility.

According to the karyotypic results of his family members, the patient inherited the der (3) from his father. Then the couple chose to get their offsprings through intracytoplasmic sperm injection based upon genetic counselling. G-banding analysis showed that the fetus inherited the der (3) from the patient, which indicated that an unbalanced paternally inherited t(Y;3) translocation existed through 3 generations in this family. Since the patient presented severe oligozoospermia, it could be speculated that the child might also suffer the fertility problems when he reaches adulthood. So if the couple intends to conceive again, preimplantation genetic diagnosis would be an ideal choice to select a normal chromosome 3 for their offsprings.

## 6 Conclusion

In conclusion, we described a severe oligozoospermic male with rare unbalanced Y; 3 translocations transmitted through 3 generations. Our study not only enriched the karyotype-phenotype correlation of Y-autosome translocations, but also strengthened the roles of molecular genetic techniques in identifying the breakpoints and regions involved, which would be helpful for such carriers with fertility problems. With increasing clinic data on Y-autosome translocations, it is anticipated that more clear karyotype-phenotype correlations would be established.

## Author contributions

**Conceptualization:** Shibo Li, Qi Xi.

**Data curation:** Hongguo Zhang.

**Formal analysis:** Hongguo Zhang, Fagui Yue.

**Funding acquisition:** Ruizhi Liu.

**Investigation:** Xiangyin Liu, Fagui Yue.

**Methodology:** Xiangyin Liu.

**Project administration:** Ruizhi Liu.

**Resources:** Yuting Jiang.

**Supervision:** Qi Xi.

**Validation:** Ruizhi Liu.

**Visualization:** Yuting Jiang, Shibo Li.

**Writing – original draft:** Shu Deng.

**Writing – review & editing:** Qi Xi.
